# A case of symptomatic tailgut duplication cyst(retro-rectal cystic hamartoma) in an adult male

**DOI:** 10.1259/bjrcr.20150422

**Published:** 2017-06-22

**Authors:** Koushik Sarkar, Pranay Kabiraj, Debasis Deoghuria, Jayati Bardhan

**Affiliations:** Department of Radiodiagnosis, Bankura Sammilani MedicalCollege, Bankura, West Bengal

## Abstract

Tailgut duplication cyst (retro-rectal cystic hamartoma) is a rare congenital developmental lesion arising from post-natal primitive gut remnants. Tailgut cysts are found more commonly in middle-aged females. It may be asymptomatic or symptomatic in complicated cases. Major differentials include epidermoid cyst, dermoid cyst and anterior meningocele. Unfortunately no radiological sign can specifically diagnose it and surgical resection and histopathology remain the cornerstone for diagnosis. Here we present a case of symptomatic tailgut duplication cyst in an adult male.

## Clinical presentation

A 47-year-old Asian male patient presented to the surgery emergency with features of large bowel obstruction and excruciating peri-anal pain for 10 days. On digital rectal examination we could palpate a large firm smooth lump behind the posterior rectal wall. The upper limit of the swelling was not palpable. The posterior rectal wall had restricted mobility and the mass obliterated the lumen of the rectum.

The general condition of the patient was poor at the time of presentation and he was severely anaemic and dehydrated. After initial resuscitation, which included correction of the anaemia, protein and electrolyte status, the necessary investigations were done. The imaging studies are described below.

## Investigations

**Figure 1.**
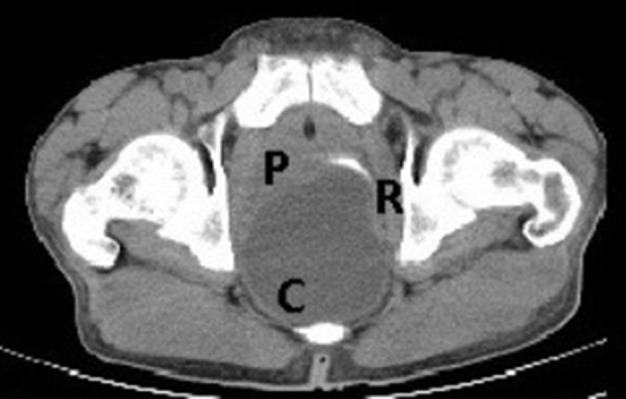
CT NC axial: a well-defined thin-walled smooth cystic SOL (C) compressing the anteriorly displaced rectum (R). UB, urinary bladder.

An ultrasonography of the lower abdomen showed a smooth cystic space-occupying lesion (SOL) with post acoustic enhancement behind the rectal wall. On non- contrast enhanced CT, a smooth-walled cystic SOL in the pre-sacral region was noted without any evidence of calcification within the lesion, thus excluding the possibility of dermoid. The bone window showed a normal sacrum without any anterior bony defect. A defect is usually present in the case of anterior meningocele. On contrast study there was almost nil enhancement of the cyst or its wall; a thick irregular enhancing wall would have supported a diagnosis of pelvic abscess. MRI showed a well-defined cystic SOL with a solid mural nodule and few thin septa within. No fistulous connection with the rectum or the spinal neural canal was found. No obvious continuity was noted with the subarachnoid space, thus excluding the diagnosis of anterior sacral meningocele. Thus considering the positive and the relevant negative points the case was provisionally diagnosed to be a case of tailgut duplication cyst or retro-rectal cystic hamartoma.

(I) Non-contrast and contrast enhanced CT—Figure 1,2. The findings are as follows: A well-defined thin-walled smooth cystic SOL(C) compressing the anteriorly displaced rectum (R). UB, urinary bladder.

(II) MRI—Figure 3, 4. The findings are as follows:Axial and sagittal *T*_2_ weighted images show the septa and a mural nodule within. The cyst wall appears to be tightly adherent to the posterior wall of the rectum (R). Urinary bladder (UB) with catheter bulb (B) in situ noted. S, sacrum; P, pubic bone.

(III) HPE—Figure 5. The findings are as follows: Section examined shows colonic tissues showing mucosa and submucosa. The mucosa contains crypts and glandular structures lined by mucus secreting tall columnar cells with basal nuclei. The sub-mucosa shows no significant histological abnormality.

**Figure 2.**
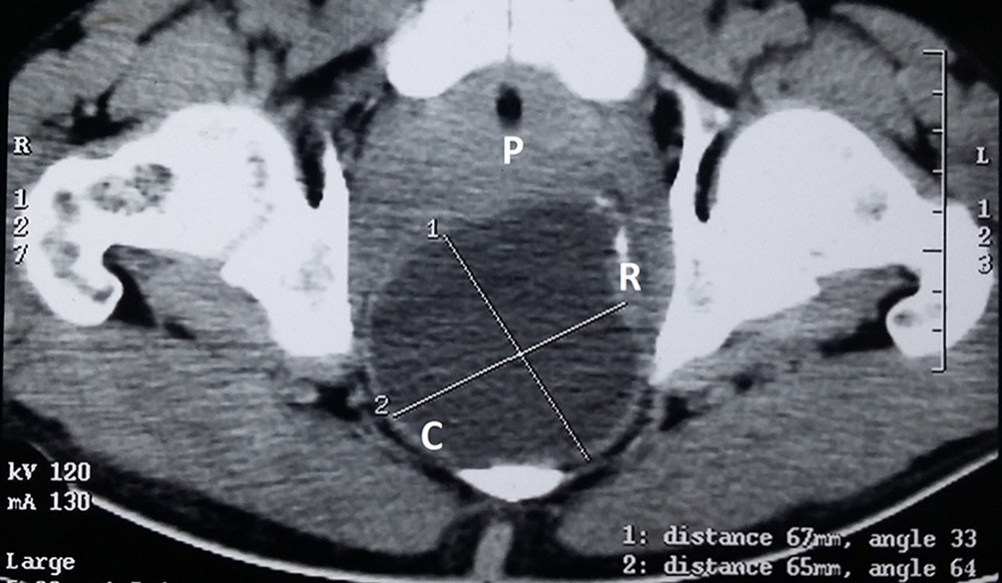
CT CE axial: A well-defined thin-walled smooth cystic SOL (C) compressing the anteriorly displaced rectum (R). UB, urinary bladder.

**Figure 3.**
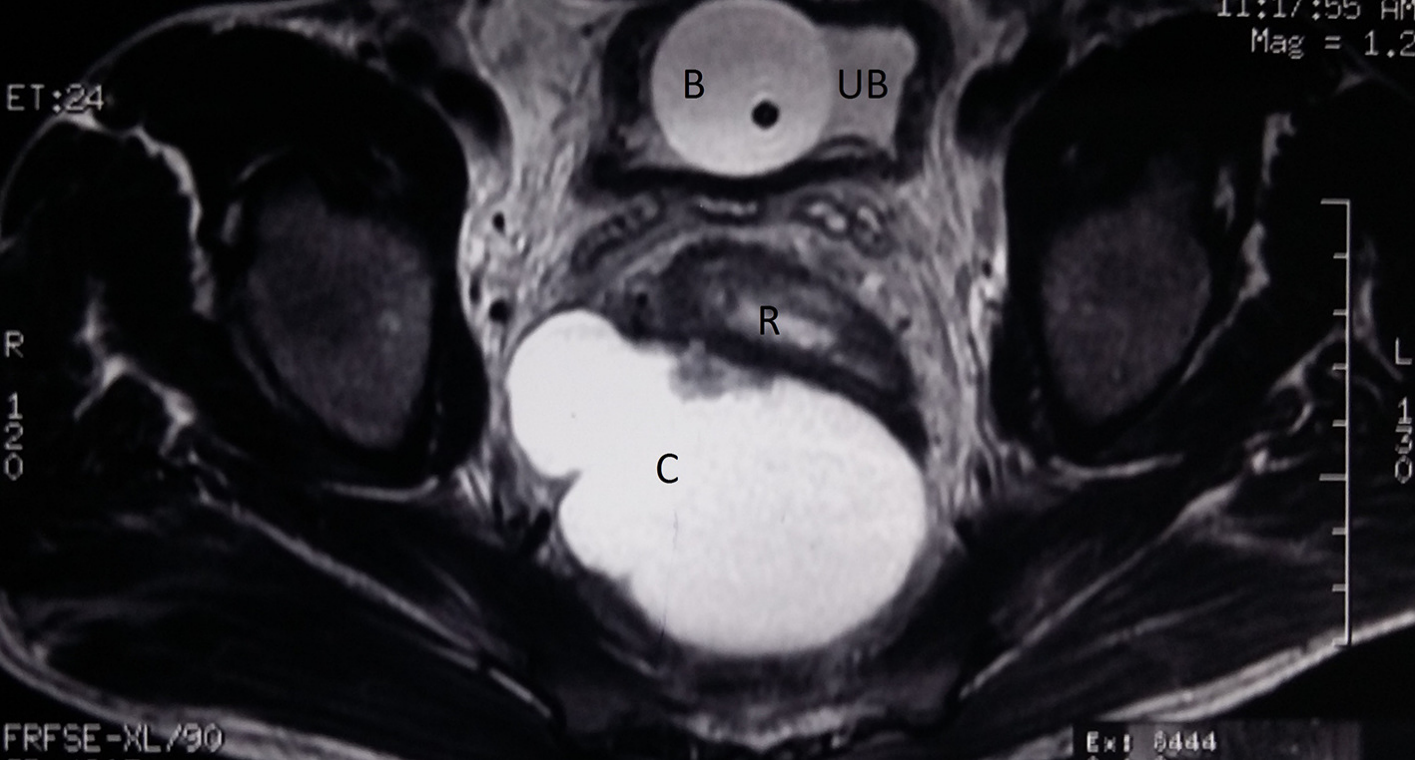
MRI axial: axial and sagittal *T*_2_ weighted images show the septa and a mural nodule within. The cyst wall appears to be tightly adherent to the posterior wall of the rectum (R). Urinary bladder (UB) with catheter bulb (B) in situ noted. S, sacrum; P, pubic bone.

**Figure 4.**
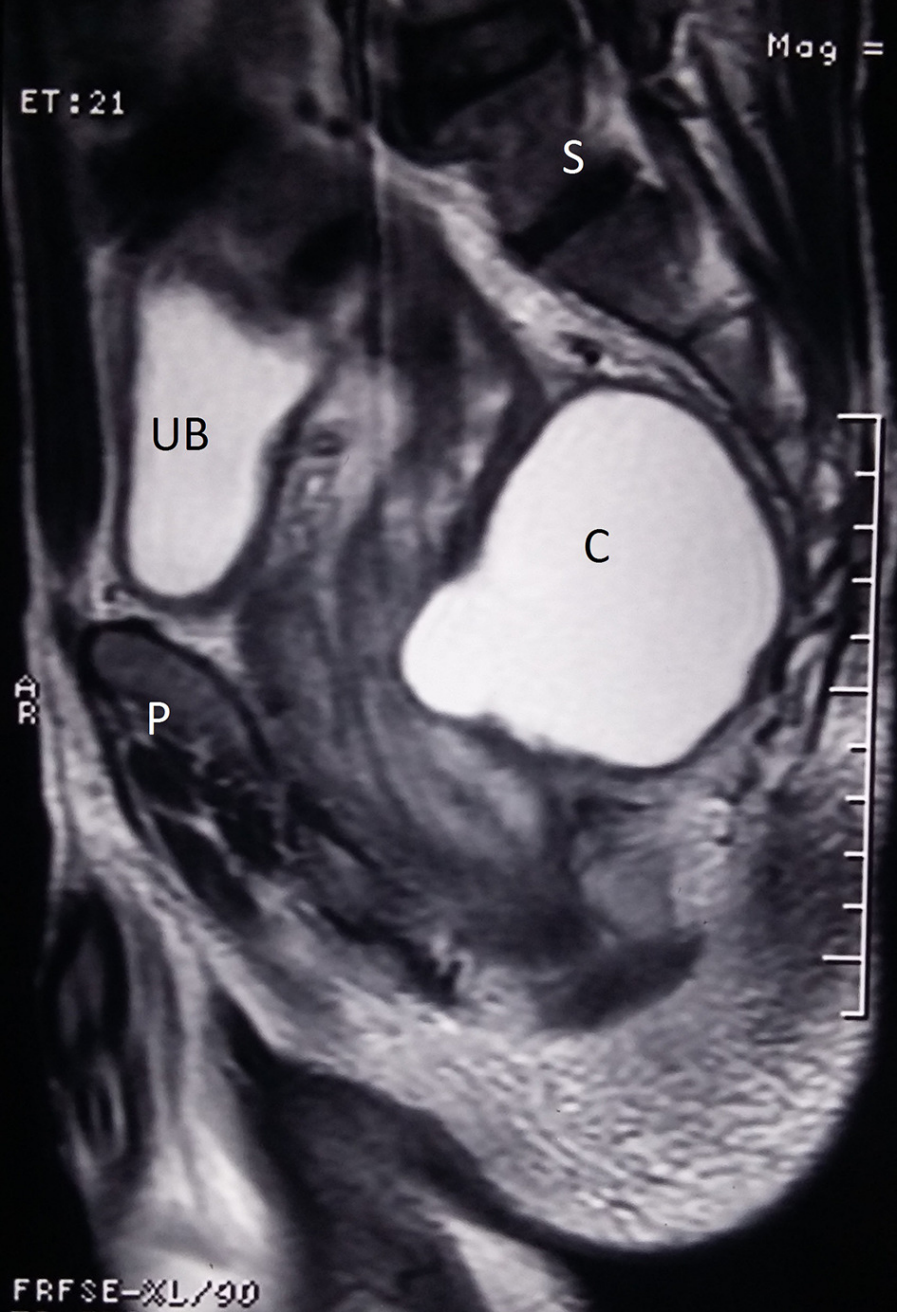
MRI sag: axial and sagittal *T*_2_ weighted images show the septa and a mural nodule within. The cyst wall appears to be tightly adherent to the posterior wall of the rectum (R). Urinary bladder (UB) with catheter bulb (B) in situ noted. S, sacrum; P, pubic bone.

**Figure 5.**
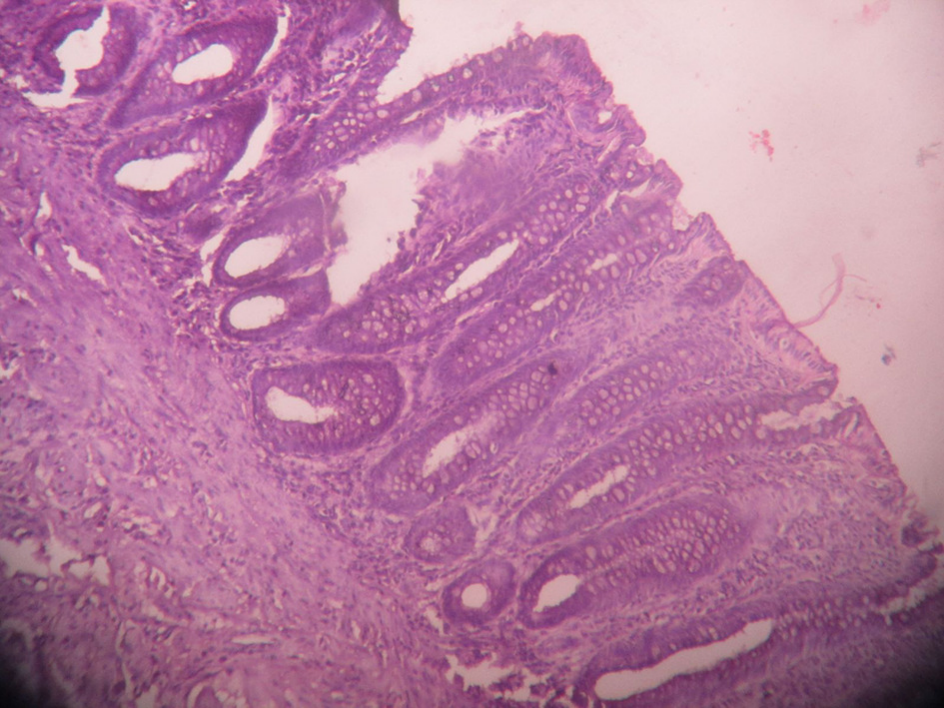
HPE: Section examined shows colonic tissues showing mucosa and sub mucosa. The mucosa contains crypts and glandular structures lined by mucus secreting tall columnar cells with basal nuclei. The sub-mucosa shows no significant histological abnormality.

## Differential diagnosis

The differential diagnoses of pre-sacral cystic SOLs include the following:

1. Dermoid cyst—these lesions are teratoma of a cystic nature containing mature tissue components like tuft of hair, cartilage or teeth. CT demonstrates areas of fat (very low Hounsfield values), calcification within the lesion.
2. Anterior sacral meningocele—it is a meningeal cyst in the pre-sacral region with congenital defect in the anterior aspect of sacrum. CT bone window shows sacral defect on the anterior aspect.
3. Epidermoid cyst—it shows heterogeneous low echogenicity on USG. On MRI there is low signal intensity on *T*_1_ weighted image and high signal intensity with islands of low intensities on *T*_2_ weighted image.
4. Tail-gut duplication cyst—the cystic nature of the mass is evident in all imaging modalities. Occasionally few septae within and the cyst appears to be dense adhered to posterior rectal wall. Diagnosis is mainly histo-pathological.
5. Ischio-rectal abscess—this is usually irregular in outline with thick enhancing wall. On MRI it shows restricted diffusion and peri-focal inflammatory changes.

## Treatment and outcome

The patient underwent exploratory celiotomy, which revealed a cystic mass, was found to be densely adherent to the posterior rectal wall. Few adhesions were seen between the cyst wall and sacrum but it yielded to blunt finger dissection. Due to the presence of dense adhesions, entire cystectomy could not be completed through the midline celiotomy incision. A trans-perineal approach was needed for complete dissection, and a part of the rectum had to be resected in order to achieve the same. Poor general condition did not allow primary anastomosis to be done and a temporary colostomy was done. Post-operative period was uneventful and the patient is now recovering well and waiting for a colostomy reversal surgery.

## Discussion

The rare recto-rectal cystic lesions in adult are mostly congenital in origin.^1^ During the fourth week of the gut development, the post anal gut or the tailgut appears which is a distal recess of the hindgut. At about 35 days of gestation, the tailgut measures the maximum about 8 mm and it usually regresses at about 56 days of gestation. Congenital cysts arise from the remnants of this tailgut.

Tailgut cysts are usually asymptomatic and most commonly found in middle-aged females of Caucasian origin. Average age of presentation is around 35 years. Symptoms may be related to the mass effect of the cyst when it becomes considerably large in size; these include rectal bleeding and altered bowel habits. Secondary infection or malignant changes may complicate the cyst.^2–4^ Adenocarcinoma of the tailgut remnant cyst has been reported in literature.^5^

Nearly all the cases reported in the literature are in female patients, but our patient was male, which makes this case a rare one. To the best of our knowledge symptomatic tailgut duplication cyst in an adult male has not been reported in literature so far. The symptoms related to this case can be explained by a super-added infection. The patient also had symptoms related to the large size of the mass. The histopathology examination confirmed the diagnosis of tailgut duplication cyst. No malignant degeneration was seen even after extensive review.

The differential diagnosis of the cystic lesions in the pre-sacral space is extensive and radiological examination can aid in diagnosis, but a definite diagnosis can only be done by surgical exploration and HPE.

## Acknowledgements

We are grateful to the patient and the editorial board of BJR for this opportunity.

## Learning points

1. Pre-sacral cystic lesions are rare developmental cysts with a wide variety of differential diagnoses.
2. No radiological sign can definitely diagnose the nature of the lesion correctly and histopathological examination remains the mainstay of evaluation.
3. Tailgut duplication cysts are usually seen in middle-aged females, but male patients can also be seen as in this case.
4. Symptomatic tailgut cysts are usually due to infection or malignant degeneration.
5. Complete excision of the cyst is required because of the risk of secondary infection or malignant changes in the residual tissue.
6. Exhaustive HPE evaluation is needed to exclude any focus of malignant tissue within the cystic lesion.

## Consent

A detailed informed consent in his own mother tongue has been obtained from the patient and the anonymity of the patient and related family members have been maintained.
